# Similarity Downselection: Finding the *n* Most Dissimilar Molecular Conformers for Reference-Free Metabolomics

**DOI:** 10.3390/metabo13010105

**Published:** 2023-01-09

**Authors:** Felicity F. Nielson, Bill Kay, Stephen J. Young, Sean M. Colby, Ryan S. Renslow, Thomas O. Metz

**Affiliations:** 1 Pacific Northwest National Laboratory, Biological Sciences Division, Richland, WA 99354, USA; felicity.nielson@gmail.com (F.F.N.); sean.colby@pnnl.gov (S.M.C.); ryan.renslow@pnnl.gov (R.S.R.); 2 Pacific Northwest National Laboratory, Advanced Computing, Mathematics, and Data Division, Richland, WA 99354, USA; william.kay@pnnl.gov (B.K.); stephen.young@pnnl.gov (S.J.Y.)

**Keywords:** conformer, downselection, graph, metabolomics, molecule, Monte Carlo, Python, sampling, structure, similarity)

## Abstract

Computational methods for creating in silico libraries of molecular descriptors (e.g., collision cross sections) are becoming increasingly prevalent due to the limited number of authentic reference materials available for traditional library building. These so-called “reference-free metabolomics” methods require sampling sets of molecular conformers in order to produce high accuracy property predictions. Due to the computational cost of the subsequent calculations for each conformer, there is a need to sample the most relevant subset and avoid repeating calculations on conformers that are nearly identical. The goal of this study is to introduce a heuristic method of finding the most dissimilar conformers from a larger population in order to help speed up reference-free calculation methods and maintain a high property prediction accuracy. Finding the set of the *n* items most dissimilar from each other out of a larger population becomes increasingly difficult and computationally expensive as either *n* or the population size grows large. Because there exists a pairwise relationship between each item and all other items in the population, finding the *set* of the *n* most dissimilar items is different than simply sorting an array of numbers. For instance, if you have a set of the most dissimilar *n* = 4 items, one or more of the items from *n* = 4 might not be in the set *n* = 5. An exact solution would have to search all possible combinations of size *n* in the population exhaustively. We present an open-source software called similarity downselection (SDS), written in Python and freely available on GitHub. SDS implements a heuristic algorithm for quickly finding the approximate set(s) of the *n* most dissimilar items. We benchmark SDS against a Monte Carlo method, which attempts to find the exact solution through repeated random sampling. We show that for SDS to find the set of *n* most dissimilar conformers, our method is not only orders of magnitude faster, but it is also more accurate than running Monte Carlo for 1,000,000 iterations, each searching for set sizes *n* = 3–7 out of a population of 50,000. We also benchmark SDS against the exact solution for example small populations, showing that SDS produces a solution close to the exact solution in these instances. Using theoretical approaches, we also demonstrate the constraints of the greedy algorithm and its efficacy as a ratio to the exact solution.

## 1. Introduction

The metabolomics analysis of biological and environmental samples can give rise to thousands of features (e.g., from LC-IMS-MS/MS or related multi-dimensional methods). To make identifications in these complex samples, the gold standard approach is to compare experimental features to a library of features created through the analysis of authentic reference materials (i.e., standards) analyzed by the same analytical platform. Due to the high complexity and/or low concentration of components, these samples are not amenable to traditional de novo structure elucidation methods (e.g., nuclear magnetic resonance). One of the current major roadblocks in metabolomics is the discrepancy in the size of identification libraries compared to the number of compounds in the molecular universe (both natural and man-made molecules), leaving metabolomics researchers in a position where samples cannot be fully characterized. Thus, unfortunately, most of the features observed in studies of complex samples remain unannotatable. This issue remains because most molecular structures are unknown and from those that are known, the set available as purchasable or easily synthesizable is limited.

A growing alternative to building identification libraries in the laboratory through the analysis of standards is to computationally predict or calculate the chemical properties and instrument responses, i.e., build in silico libraries. As computational resources, instrument physics understanding, and algorithm accuracy have improved, more groups have begun exploring “reference-free” computational metabolomics methods. Common methods for in silico library building include cheminformatics/QSAR, AI/ML, and quantum chemical calculations, and consider features/properties such as retention time, drift time/collision cross section, monoisotopic signatures, ionization adduct types, infrared spectra, mass fragmentation patterns, and more.

For all methods, but especially quantum chemical calculations, obtaining a good representation of the geometries of the molecules, i.e., conformers, is critical for increasing accurate libraries. Conformers are molecules with identical bonds and atoms but different structures (i.e., geometric arrangements of the atoms relative to each other). For each molecule, there is an infinite number of conformers, but only a small set have low Gibb’s free energy and are therefore probable to find under experimental conditions. A nontrivial problem is creating sets of conformers and selecting the critical few that should be used for the final property calculations. Thus, several groups have striven to create algorithms and automated pipelines in order to smartly sample conformers sets: e.g., clustering methods [1], randomly sampling (with or without simulated annealing) [2,3,4], or selecting low energy sets from classical or semiempirical molecular modeling [5,6]. Part of the goal of downselecting to a smaller set of conformers is to save on the relatively high computational cost when performing quantum chemical calculations. In a recent study, we found that by selecting the two most dissimilar and single most similar conformers from simulated annealing runs prior to performing density functional theory calculations, we could save substantial computational time while preserving property calculation accuracy [6]. However, that method was not scalable, and so we endeavored to find a new subsetting method that could work well in high-throughput pipelines and consider thousands of conformers.

There exists a large and historical body of algorithms and their implementations for searching, sorting, and clustering data based on distance or similarity. Popular algorithms include beam search and K-means clustering [7,8,9,10], used for finding the target result by following the most promising nodes (as determined by an evaluation function *f(n)*) and for grouping data by similarity to a number of selected data points or nodes, respectively. One similarity-comparison problem involves choosing the set of the *n* most dissimilar items from a larger population of size *N*, in which there exists a pairwise relationship between each item and all other items. There exist several older algorithms in the literature to solve this type of problem [11,12,13] but none are available as open-source Python packages. The solution to this dissimilarity-set problem is useful in chemistry and biology, for instance, for finding the most geometrically dissimilar sets of conformers (or molecular structures) to efficiently span conformational space and eliminate redundant structures. The use of root-mean-square deviation (RMSD) of atomic positions for selecting sets of conformers has been used by many groups [2,5,14,15,16].

Finding the exact solution to the most dissimilar set problem becomes intractably computationally expensive (super-exponentially complex) as the population size *N* grows large, and it is most expensive when *n* = *N*/2, according to the binomial coefficient

(1) $$\left(\begin{array}{c} N\\ n \end{array}\right)=\frac{N!}{\left(N-n\right)!n!}$$

Additionally, if the set *n* = 4 is found, one or more of its items might not be a member of the set *n* = 5. Verifying that the exact solution has been found requires exhaustively searching all possible combinations for a given set size. Finding the exact solution quickly becomes intractable for classical computers. When considering a population of 50,000 items, for example, finding the most dissimilar set of *n* = 3 items would require searching over 20 trillion unique combinations (2.083208335 × 10^13^ to be exact). However, if one assumes all members of the set *n* are also members of the set *n* + 1, then an approximate solution that is sufficiently close to the exact solution can be determined in a “greedy” fashion. We introduce a heuristic algorithm implemented in Python, similarity downselection (SDS), that finds the subset of the *n* most dissimilar items from a large population. As described, this type of algorithm is critical for helping to accelerate reference-free metabolomics methods for computing in silico libraries, but it is also generally applicable to other similar subsetting problems across all domains of science. SDS is generalizable to any application where the data can be represented as arrays whose elements are the pairwise relationships between each item and all other items in the population. We include a brief description of an example application on molecular conformer selection, and benchmark SDS against both a Monte Carlo sampling method and the exact solution. In addition, we demonstrate the constraints and efficacy of the algorithm using triangle approximations and ratios (Supplementary Information).

## 2. Application: Molecular Conformer Sampling

**Figure 1:**
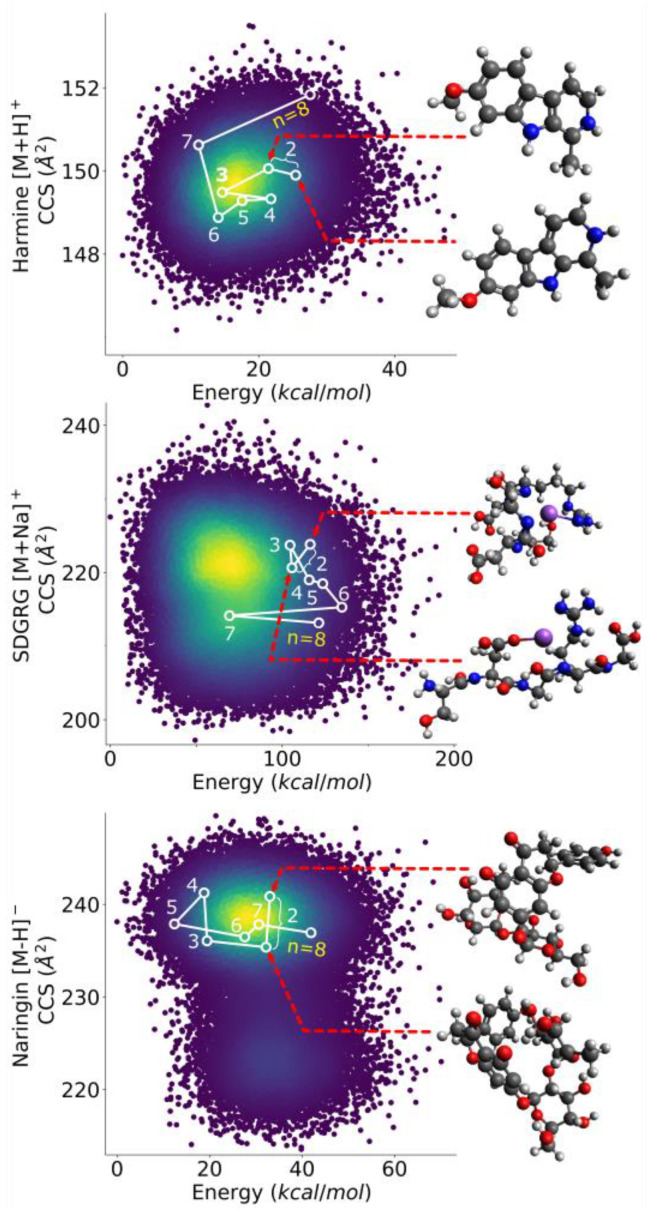
Demonstration of SDS choosing the 8 most mutually dissimilar conformers for Harmine [M+H]^+^, SDGRG [M+Na]^+^, and Naringin [M−H]^−^, showing the structure of the three most dissimilar conformers for each. SDS works iteratively by finding the set *n* + 1 by building off the set *n*.

It was previously found in ISiCLE that the Boltzmann-weighted average of the two most dissimilar and one most similar conformer chosen out of 10 AMBER simulated annealing cycles saved ~70% time while still being within >99% agreement with the full conformer sampling, as compared to the Boltzmann-weighted average of every conformer out of every cycle. This is thought to be because choosing the most dissimilar conformers spans conformational space with fewer conformers. It should be noted that choosing the *n* conformers most dissimilar from each other is not the same as choosing the *n* conformers most dissimilar from the whole population. This is because two conformers can be similar to each other and dissimilar from the average population at the same time. Finding the two most dissimilar conformers is trivial, but finding the subsets of three or more is not. As part of a larger analysis to assess the validity of various conformer selection techniques [6], a method was needed to efficiently find the subset of the *n* most dissimilar molecular conformers out of a set of 50,000 conformers (where 1 < *n* < 50,000). These conformers were generated by a modified ISiCLE pipeline on a set of 18 small molecules with mass ranges of ~100–700 Da, as described previously [6]. The goal was to efficiently span conformer space by downselecting from a larger population to the most structurally dissimilar conformers. SDS was developed to this end and was proven to outperform a proposed Monte Carlo method in both time and accuracy, as shown in Section 4 below. Figure 1 demonstrates the 8th dissimilar set being iteratively chosen by SDS on Harmine [M+H]^+^, SDGRG [M+Na]^+^, and Naringin [M−H]^−^, and plotted in CCS vs. energy space.

The dissimilarity between two conformers was measured as the average pairwise RMSD between corresponding atoms, calculated using OpenBabel (v 2.4.1) [17,18]. The method for finding the *nth* dissimilar set needed to be efficient and applicable to any small molecule. The section below provides a description of the SDS algorithm.

## 3. Similarity Downselection Python Module

SDS implements a heuristic algorithm for finding the set of *n* items most dissimilar from each other out of a larger population. The algorithm is greedy, making the optimal choice during each iteration, where each iteration finds the set *n* + 1 by building off the set *n*. SDS is freely available as a Python module on GitHub at https://github.com/pnnl/sds (accessed on 1 December 2022) and in the Supplementary Materials. Below, we provide short descriptions of how the algorithm works using arrays and, alternatively, using node/graph theory.

### 3.1. Algorithm Description

The individual items in a population are represented as arrays whose elements contain floating point values of the pairwise relation (e.g., RMSD or other dissimilarity metric) between the given item and all other items in the population. The first element of all arrays is reserved for the pairwise relation to the first item, the second element to the second item, and so forth until an *NxN* matrix is formed, where *N* is the size of the total population, the *ith* row is the array of the *ith* item, and *N_ij_* contains the pairwise relation between items *i* and *j*. Since *N_ij_* = *N_ji_*, the matrix is symmetric across the diagonal.

**Figure 2:**
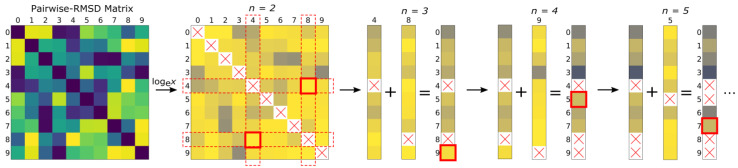
Illustration of the similarity downselection algorithm. The natural log is taken on a square matrix containing the pairwise-similarity relations of the items in the full population. The two most dissimilar items (i.e., most dissimilar subset *n = 2*) are found and their arrays summed to find the third most dissimilar item (i.e., subset *n* = 3). Successive most dissimilar subsets are iteratively found by adding the array of the most recently found item to the summation array and taking the index of the largest (or smallest) value. Items already selected cannot be selected again and are represented as nan in the summation array.

The algorithm first selects the two items that have the largest pairwise value between them. This is the exact subset of *n* = 2 most dissimilar items. To find the subset *n* = 3, the natural log of the first two arrays (corresponding to the first two items) are summed element-wise to create a new summation array. The index of the largest value in the summation array is the index of the third most dissimilar item. The natural log of the array corresponding to the third item is then added to the summation array to yield the index of the fourth most dissimilar item. Successive subsets *n* = 5, 6, 7, …, *N*, are achieved in the same manner, selecting the item corresponding to the index of the highest value, as shown in Figure 2.

Items could be multiplied into a multiplication array instead of log-summed, but it was found the product of floating-point numbers quickly exceeds machine precision (10×10^323^ in our setup) after about 10,000 items, so log-summing was used instead. Effectively, log-summing (or multiplying) rewards items that have a large value across all arrays by making its numerical representation larger and punishes items that have even one significantly small pairwise relation with another item by making its numerical representation smaller. *N* can be very large, theoretically indefinite, and limited only by machine precision and memory. The population used for the original implementation, as discussed in Section 3, contained 50,000 items.

### 3.2. Problem and Algorithm Description Using Graph Theory (Nodes and Edges)

Like the traveling salesman problem, the longest path problem (LPP), and many other problems found in graph theory, the items in the most dissimilar set problem (MDSP) can be thought of as nodes. Like the LPP for weighted complete graphs (where each node is connected to every other node and the edges are assigned weights), the MDSP seeks to find the nodes that will maximize the total distance. More specifically, the MDSP must find the subset of size *n* that will yield a maximum distance. In graph theory, this makes the MDSP more general than the LPP because the LPP is a special case when *n* = *N*. In MDSP permutation, unlike LPP permutation, the order the nodes are visited does not matter. For example, when the target set size is the full population size (*n =* N), the solution to the MDSP is trivially the full population. In contrast, the solution for the LPP has not only not been found, it has not even been searched for. Additionally, while problems like the LPP require the total distance be calculated by the simple path traveled between nodes (only two connecting edges per node: entering and exiting), the MDSP takes into account all pairwise edges in the solution set exactly once. In other words, the exact solution to MDSP can and must travel all pairwise edges in the chosen set and does not double count edges that have already been traveled.

SDS uses a one-dimensional representation to inform its decision traversing the graph. The algorithm starts by finding the two nodes most distant from each other (two nodes with the highest weight assigned to their connecting edge). It then takes the one-dimensional representation of one of the two nodes and log-sums its weighted edges (distances) with the one-dimensional representation of the second node. This creates a new one-dimensional representation where all previously chosen nodes have zero distance and the node with the furthest distance (highest log-sum) is the next node chosen on the graph.

## 4. Benchmarking

### 4.1. Performance against a Monte Carlo Method

**Figure 3:**
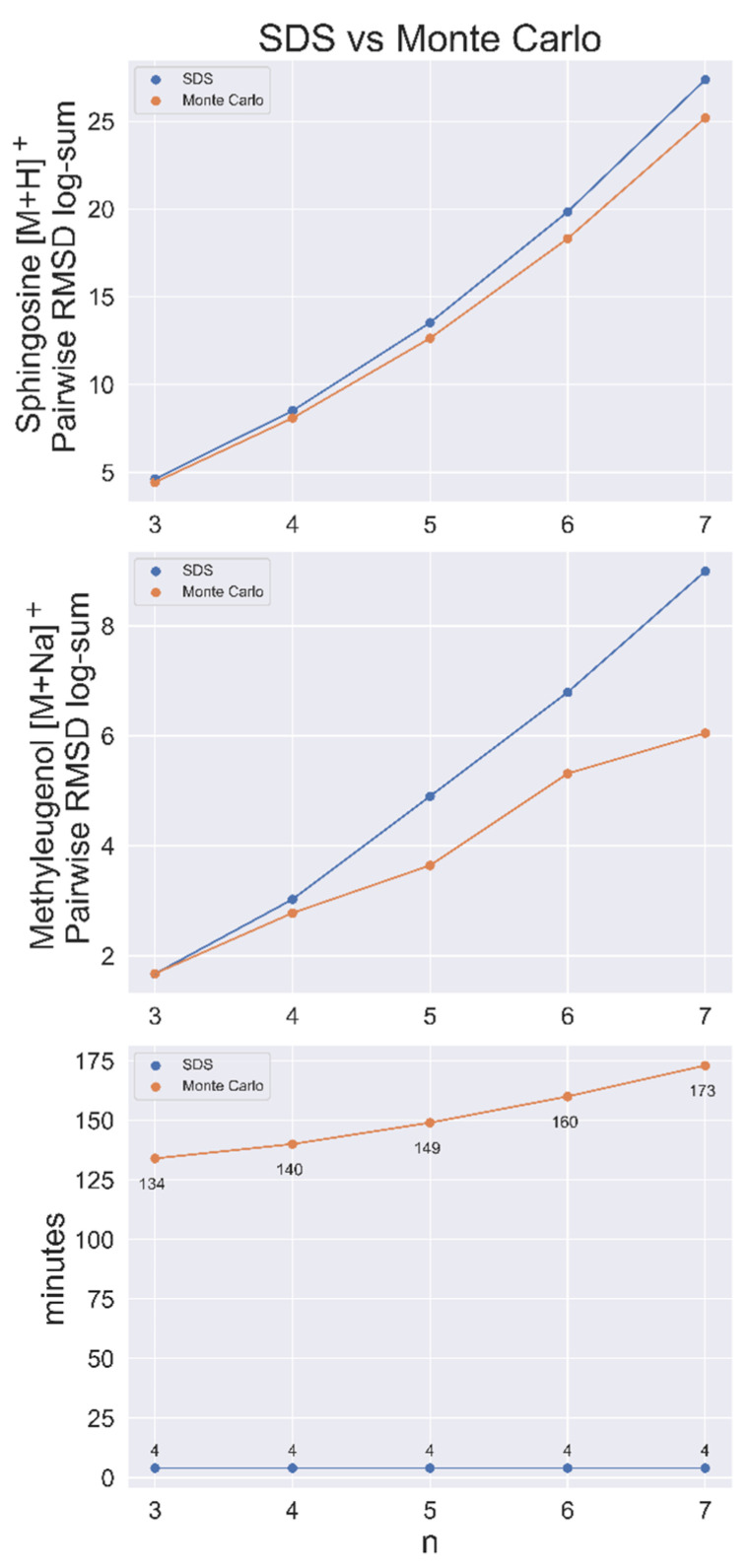
SDS benchmarked against a Monte Carlo (MC) sampling method for sphingosine [M+H]^+^ and methyleugenol [M+Na]^+^ with conformer populations of 50,000. Top and middle, the conformer RMSD log-sum (a metric of the dissimilarity of the set) for SDS and the largest RMSD log-sum found via the MC method for set size *n*. Bottom, search time per node for both methods. Time includes the (approximate) 3 min to load the pairwise RMSD matrix.

SDS was shown to be faster and produce more dissimilar sets than a Monte Carlo (MC) sampling method in a contest to find the most dissimilar sets of *n* = 3–7 out of a population of 50,000 conformers for sphingosine [M+H]^+^. MC sampling was run for 1,000,000 iterations for each *n*-sized set, with each taking more than 2 h to complete. After loading the data matrix, which required about 3 min, the heuristic algorithm found all sets in <1 min. SDS also had a greater RMSD log-sum (total distance between nodes) for every set size, as shown in Figure 3, indicating that it was closer to the exact solution than the MC method every time.

This benchmarking analysis was applied again to 50,000 conformers of methyleugenol [M+Na]^+^, with similar results. Here, MC performed better than SDS at *n* = 3 by a small margin (Figure 3). SDS ran the complete search for every possible set of 1 < *n* < 50,000 in approximately 7 min, including the approximate 3 min required to load the matrix.

### 4.2. Performance against the Exact Solution

**Figure 4:**
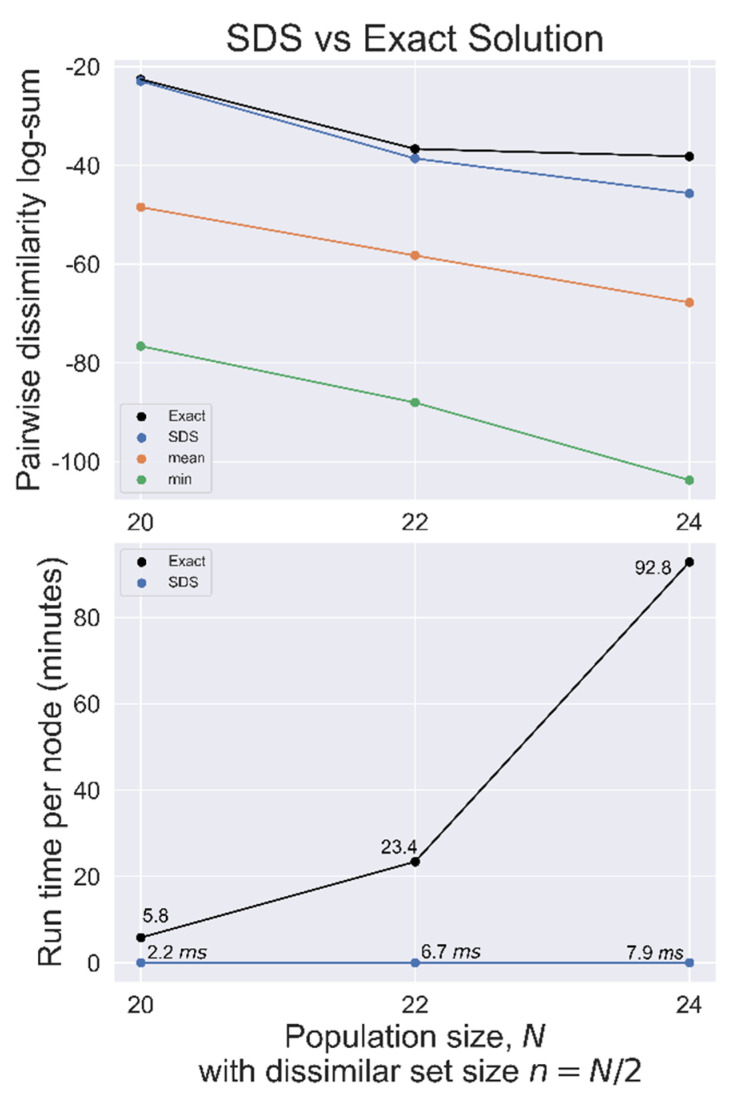
SDS benchmarked against the exact solution used on randomly generated datasets with population size *N*, searching for the most dissimilar set of size *n* = *N*/2. **Top**, total pairwise dissimilarity for the exact solution, SDS, mean, and minimum (most similar) sets. **Bottom**, search time per node for both methods.

SDS was benchmarked against the exact solution for *N =* 20, 22, and 24 with *n = N*/2 used on randomly generated datasets, as summarized in Figure 4. In each case, the SDS solution had a total distance closer to the exact solution distance than the mean set, indicating a good heuristic solution.

In our setup, we estimate that it would require over 72 node hours to find the exact solution for a population even as small as *N* = 30 with *n* = 15 while SDS would find a heuristic solution in a fraction of a second. Despite the extensive work in this type of problem, we are unaware of any approaches which have provided provable performances guarantees. In the Supplementary Information, we show that if the dissimilarity is measured as the product of Euclidean distances, then there are at least some performance guarantees for the greedy heuristic.

### 4.3. Comparing Computational Costs of Calculating Pairwise Relations

SDS requires the pairwise relation matrix to be calculated in advance. Depending on the application and what set size is being searched for, initially this may falsely appear to reduce the cost-effectiveness. Because the same pairwise relations would have to be calculated for both the MC method and the exhaustive search, the total computational cost is equal to the cost for SDS, assuming each relation is efficiently calculated only once during each method. If MC fails to consider every possible pairwise relation and is computing them on the fly, then there would be fewer pairwise relations to compute, but this would be the same as creating a randomly selected subset of data and running MC searches when SDS could have been run on the subset just as well, underscoring the benefit of SDS.

## 5. Conclusions

We have introduced new software written in Python that implements a heuristic algorithm for finding the set of *n* items most dissimilar from each other. We have demonstrated its efficacy and efficiency in benchmarks against a Monte Carlo method, and the exact solution and provided mathematical evidence for the limitations of the algorithm (Supplementary Information). SDS, freely available at https://github.com/pnnl/sds (accessed on 1 December 2022) with instructions for running on the command line or a step-by-step Jupyter Notebook tutorial, has application in molecular conformer selection when using computational methods to predict properties in “reference-free” metabolomics studies, but also has potential application in searches for the *n^th^* most dissimilar set in generalized datasets.

## Data Availability

The source code for SDS can be found as Supplementary Material.

## Supplementary Materials

The following supporting information can be downloaded at: https://www.mdpi.com/2218-1989/13/1/105/s1, SDS code (Nielson_etal_2021.zip). References [19,20,21,22,23,24,25] are cited in the supplementary materials.
