# Correction: Nielson et al. Similarity Downselection: Finding the *n* Most Dissimilar Molecular Conformers for Reference-Free Metabolomics. *Metabolites* 2023, *13*, 105

**DOI:** 10.3390/metabo13111158

**Published:** 2023-11-17

**Authors:** Felicity F. Nielson, Bill Kay, Stephen J. Young, Sean M. Colby, Ryan S. Renslow, Thomas O. Metz

**Affiliations:** 1 Pacific Northwest National Laboratory, Biological Sciences Division, Richland, WA 99354, USA; felicity.nielson@gmail.com (F.F.N.); sean.colby@pnnl.gov (S.M.C.); ryan.renslow@pnnl.gov (R.S.R.); 2 Pacific Northwest National Laboratory, Advanced Computing, Mathematics, and Data Division, Richland, WA 99354, USA; william.kay@pnnl.gov (B.K.); stephen.young@pnnl.gov (S.J.Y.)

## 1. Error in Figure

There were missing figures and associated legends for Figure 3 and Figure 4 as published due to a publication error [1]. Figure 3 and Figure 4 appear below. 

## 2. Text Correction

There was an error in the original publication [1]. The figure citation number was wrong because of the missing of Figure 3 and Figure 4.

A correction has been made to

1. Section 4.1, First Paragraph and Second Paragraph:

SDS was shown to be faster and produce more dissimilar sets than a Monte Carlo (MC) sampling method in a contest to find the most dissimilar sets of *n* = 3–7 out of a population of 50,000 conformers for sphingosine [M+H]^+^. MC sampling was run for 1,000,000 iterations for each *n*-sized set, with each taking more than 2 h to complete. After loading the data matrix, which required about 3 min, the heuristic algorithm found all sets in <1 min. SDS also had a greater RMSD log-sum (total distance between nodes) for every set size, as shown in Figure 3, indicating that it was closer to the exact solution than the MC method every time.

This benchmarking analysis was applied again to 50,000 conformers of methyleugenol [M+Na]^+^, with similar results. Here, MC performed better than SDS at *n* = 3 by a small margin (Figure 3). SDS ran the complete search for every possible set of 1 < *n* < 50,000 in approximately 7 min, including the approximate 3 min required to load the matrix.

2. Section 4.2, First Paragraph:

SDS was benchmarked against the exact solution for *N =* 20, 22, and 24 with *n = N*/2 used on randomly generated datasets, as summarized in Figure 4. In each case, the SDS solution had a total distance closer to the exact solution distance than the mean set, indicating a good heuristic solution.

The authors state that the scientific conclusions are unaffected, and we acknowledge that these figures were part of the original review. This correction was approved by the Academic Editor, and have already been approved by the reviewers. The original publication has also been updated.

## Figures and Tables

**Figure 3:**
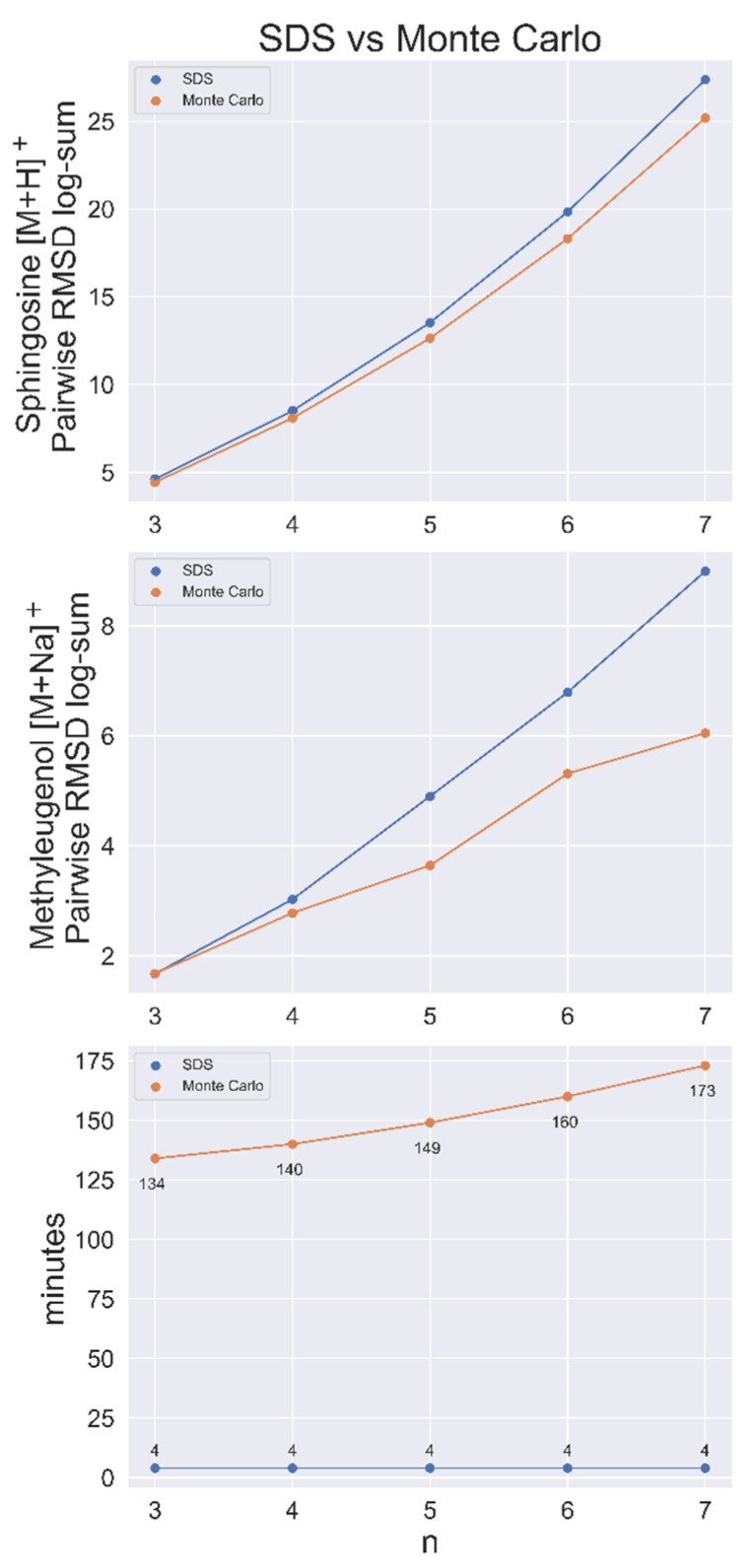
SDS benchmarked against a Monte Carlo (MC) sampling method for sphingosine [M+H]^+^ and methyleugenol [M+Na]^+^ with conformer populations of 50,000. Top and middle, the conformer RMSD log-sum (a metric of the dissimilarity of the set) for SDS and the largest RMSD log-sum found via the MC method for set size *n*. Bottom, search time per node for both methods. Time includes the (approximate) 3 min to load the pairwise RMSD matrix.

**Figure 4:**
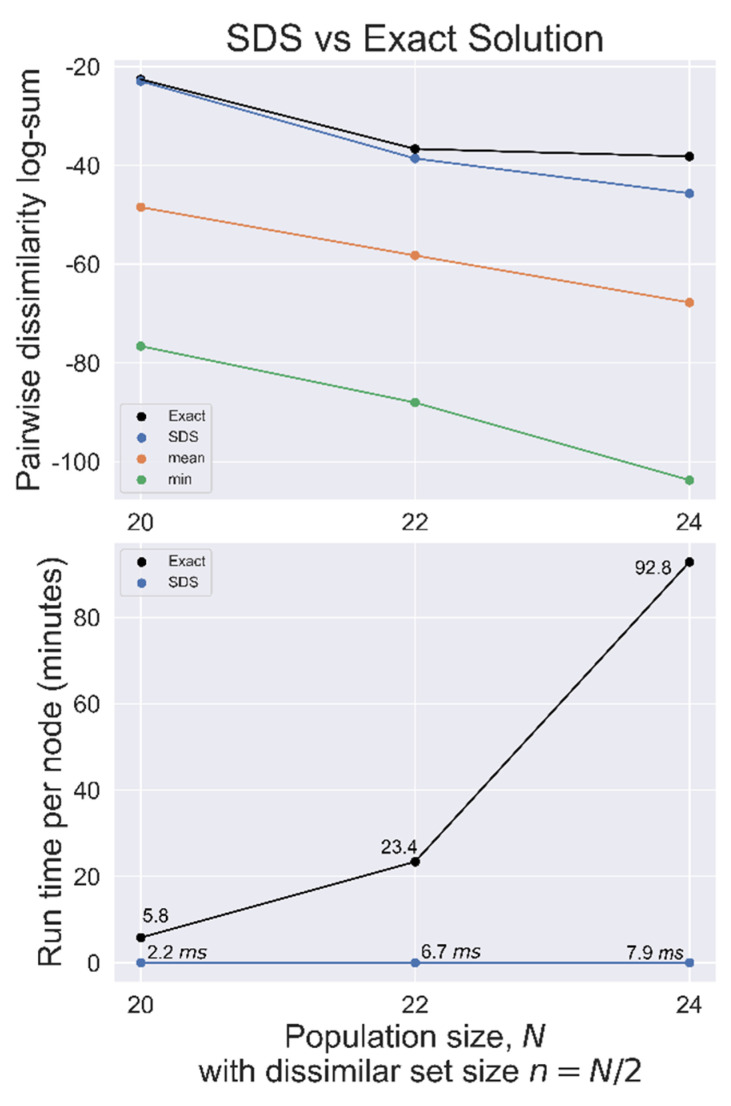
SDS benchmarked against the exact solution used on randomly generated datasets with population size *N*, searching for the most dissimilar set of size *n* = *N*/2. **Top**, total pairwise dissimilarity for the exact solution, SDS, mean, and minimum (most similar) sets. **Bottom**, search time per node for both methods.
