# Supplementary material for: Similarity Downselection: Finding the n Most Dissimilar Molecular Conformers for Reference-Free Metabolomics
Source: Metabolites. 2023 Jan 9;13(1):105. doi: 10.3390/metabo13010105 (PMC9864474; doi:10.3390/metabo13010105)
Supplement: Supplementary file 1 [file metabolites-13-00105-s001.zip › metabolites-1704187-supplementary/Nielson_etal_2022_SDS_Supplemental_final_7Oct2022.pdf]

Supplementary Information

Similarity Downselection: Finding the n Most Dissimilar Molecular Conformers for Reference-free Metabolomics

Felicity F. Nielson [1], Bill Kay [2], Stephen J. Young [2], Sean M. Colby [1], Ryan S. Renslow [1] and Thomas O. Metz [1,*]

[1] Pacific Northwest National Laboratory, Biological Sciences Division, Richland, WA 99354, USA
[2] Pacific Northwest National Laboratory, Advanced Computing, Mathematics, and Data Division, Richland, WA 99354, USA
* Correspondence: thomas.metz@pnnl.gov

Greedy Approximation to the Metric Dissimilarity Problem

Broadly speaking, our goal is to efficiently identify a subset of k conformers from a much larger set of conformers in order to capture the total range of likely geometries of the conformers. More formally, if our set of conformers is $X = \{x_1, \dots, x_n\}$ and the dissimilarity between x_i and x_j is given d_{ij}, we wish to maximize some measure of total dissimilarity $f(S)$ over all subsets of size k from X. Historically, this total dissimilarity function f is measured by averaging the dissimilarity over all pairs of elements in S. However, in practice, we would also like for these dissimilarity sets to be an efficient representation of the range of likely geometries. That is, we are willing to trade some fidelity in the representation of the conformers for a smaller set of representatives, more formally, if $|T| < |S|$ are such that $f(T)$ and $f(S)$ are approximately the same, we will prefer T to S. In order to capture this preference, we replace the sum over with pairwise similarities with the product, i.e.

$$f(S) = \prod_{\substack{i,j \in S \\ i \neq j}} d_{ij}.$$

While at first glance this would seem to add significant computational complexity, by considering

$$\log\big(f(S)\big) = \sum_{\substack{i,j \in S \\ i \neq j}} \log\left(d_{ij}\right),$$

we can preserve the same computational performance. By picking a sufficiently large constant C and considering a weighted graph with edge weights $w_{ij} = C + \log\big(d_{ij}\big)$, this can be viewed as equivalent to identify the clique of size k with the maximum total edge weight, known as the MaximumeEdgeWeightedClique [1] or the MaximumDiversity [2] problem. Unfortunately,

this problem is, in general, $\mathcal{NP}$-complete and hence computationally intractable [3]. As such, a variety of approaches have been to developed to solve instances of these problems, including integer and mixed-integer programming formulations [6,7], facet-generation algorithms [4], and various heuristics [1,2,5].

Despite the extensive work in this problem, we are unaware of any approaches which have provided provable performances guarantees. In this **Supplementary Information**, we show that if the dissimilarity is measured as the product of Euclidean distances, then there are at least some performance guarantees for the greedy heuristic. As the RMSE is an Euclidean distance up to a multiplicative factor, this is directly applicable to our approach of identifying dissimilar conformers detailed above.

1. Approximating Triangles

Although determining the maximum dissimilarity of a collection of objects is in general quite challenging, the addition of metric space constraints has the potential to provide a means of significantly reducing the computational challenges in the problem. In particular, the underlying metric space imposes significant constraints on the configurations of the points. In order to illustrate this, in this section we delve in to the problem of identifying the maximum dissimilarity triangle for a collection of points in an arbitrary Euclidean space.

Before proceeding further it is helpful to consider some simplifying assumptions for the triangle case. The first, and the most obvious, is that the ratio between the dissimilarity of a point set and the greedy approximation is invariant under rotations, translations, and scaling of the ambient space. In particular, we can assume the largest distance in the point set of interest is 1 and it occurs between a point at the origin and a point aligned with the unit basis vector e_1. Thus the question of approximation ratio can be reduced to considering points in the intersection of the spheres of radius of 1 surrounding the origin and e_1, that is, points in $B(\vec{0},1) \cap B(e_1,1)$.

This space can be further reduced by assuming a particular value for the greedy solution. In particular, if the greedy heuristic results in a triangle with dissimilarity of s, then we can further restrict our attention the points $\vec{x}$ such that $\text{dist}(\vec{x},\vec{0})\text{dist}(\vec{x},e_1) \leq s$. This leads to the final reduction, we may restrict our attention to the geometry of 3-space when determining the approximation ratio of the greedy algorithm. Specifically, since the worst-case triangle lies in a 2-dimensional subspace and the line between $\vec{0}$ and e_1 defines a 1-dimensional space, it is sufficient to consider examples in 3-space. To that end, for any $s \in (0,1]$ we define

$$S(s) = \{(x,y,z): x^2+y^2+z^2 \leq 1, (1-x)^2+y^2+z^2 \leq 1, (x^2+y^2+z^2)((1-x)^2+y^2+z^2) \leq s^2\}.$$

The approximation ratio of the greedy heuristic for the triangle is then given by $\max_{s\in(0,1]} \frac{f(s)}{s}$, where $f(s)$ is the maximum dissimilarity of a triangle in $S(s)$ where every edge length is at most 1. It is worth noting that by assumption $s \leq f(s) \leq 1$, with $f(s) = 1$ if and only if there is an equilateral triangle of side length 1 in $S(s)$.

Before turning to our results, it is helpful to explore the geometry of $S(s)$. In particular, letting $r^2 = y^2 + z^2$ in the definition of $S(s)$, it is obvious that the boundary of $S(s)$ is formed by rotating the function $r(x)$ about the x-axis where

$$r^2(x) = \min\left\{1 - x^2, 1 - (1-x)^2, \max\left\{0, -\frac{1}{4} - \left(x - \frac{1}{2}\right)^2 + \sqrt{s^2 + \left(x - \frac{1}{2}\right)^2}\right\}\right\}.$$

In Fig. S1, we provide a depiction of $r(x)$ for various choices of s to illustrate the geometry of the region $S(s)$. We note that if $s < \frac{1}{4}$, the region $S(s)$ is disconnected and the x coordinates are restricted to lie in the region $\left[0, \frac{1-\sqrt{1-4s}}{2}\right] \cup \left[\frac{1+\sqrt{1-4s}}{2}, 1\right]$.

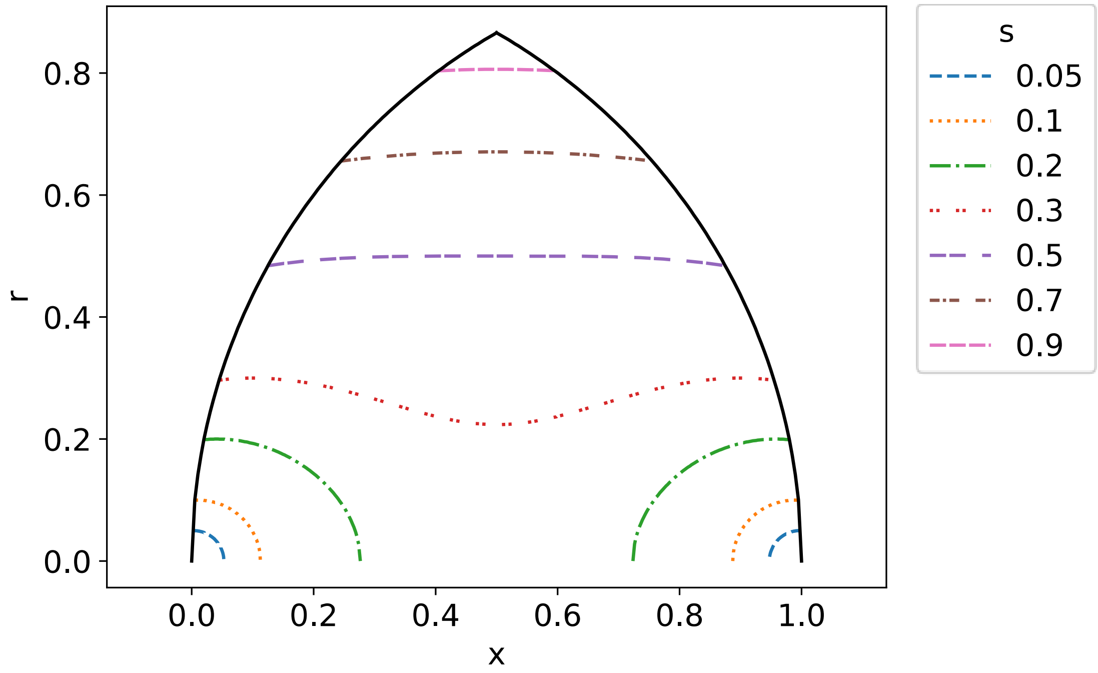

Figure. S1 Illustration of the boundary of $S(s)$ for various values of s. *The black lines correspond to the restriction that the points must be at most distance 1 from* $(0,0,0)$ *and* $(1,0,0)$*. The colored lines correspond to the restriction on the point set imposed by the greedy heuristic identifying a triangle with dissimilarity* s*.*

Looking at Fig. S1 it is unsurprising that if s is sufficiently larger, then $S(s)$ contains three points that form an equilateral triangle of side-length 1. In fact, the following result shows that we only need s to be slightly larger than $\frac{1}{2}$.

Proof. We first consider the case where the region $S(s)$ is connected, i.e. $s \geq \frac{1}{4}$. To this end, we examine family of equilateral triangles defined by $\epsilon \in \left[0, \frac{1}{2}\right]$ and $y, z \geq 0$,

$$\begin{aligned} p_1 &= \left(\frac{1}{2}, \sqrt{s-\frac{1}{4}}, 0\right) \\ p_2 &= \left(\frac{1}{2}-\epsilon, -y, z\right) \\ p_3 &= \left(\frac{1}{2}+\epsilon, -y, -z\right) \end{aligned}$$

We first note p_1 is well defined if $s \geq \frac{1}{4}$, and that $\operatorname{dist}\big((0,0,0), p_1\big)^2 = \operatorname{dist}\big((1,0,0), p_1\big)^2 = s$, so $p_1 \in S$. Since (p_1, p_2, p_3) defines an equilateral triangle with side length 1, we have that

$$\begin{aligned} 1 &= 4\epsilon^2 + 4z^2 \\ 1 &= \epsilon^2 + \left(\sqrt{s-\frac{1}{4}} + y\right)^2 + z^2. \end{aligned}$$

Thus $z = \sqrt{\frac{1}{4} - \epsilon^2}$ and $y = \frac{\sqrt{3}}{2} - \sqrt{s - \frac{1}{4}}$. As $\epsilon \in \left[0, \frac{1}{2}\right]$ and $s \geq \frac{1}{4}$, both y and z are well defined. Thus it suffices to show that $p_2, p_3 \in S$.

As both S and $\{p_2, p_3\}$ are symmetric in the plane defined by $x = \frac{1}{2}$, in order to show that $p_2, p_3 \in S$ it suffices to show that

$$\begin{aligned} \left(\frac{1}{2}+\epsilon\right)^2 + y^2 + z^2 &\leq 1 \qquad \text{and} \\ \left(\left(\frac{1}{2}+\epsilon\right)^2 + y^2 + z^2\right)\left(\left(\frac{1}{2}-\epsilon\right)^2 + y^2 + z^2\right) &\leq s^2 \end{aligned}$$

Rearranging, yields that

$$y^2 + z^2 \leq \min\left\{\frac{3}{4} - \epsilon - \epsilon^2, -\frac{1}{4} - \epsilon^2 + \sqrt{\epsilon^2 + s^2}\right\}$$

As $z^2 = \frac{1}{4} - \epsilon^2$, this is equivalent to

$$y^2 \leq \min\left\{\frac{1}{2} - \epsilon, -\frac{1}{2} + \sqrt{\epsilon^2 + s^2}\right\}.$$

Thus S contains an equilateral triangle for a given s if there exists an $\epsilon \in \left[0, \frac{1}{2}\right]$ such that

$$\frac{1}{2} + s - \sqrt{3s - \frac{3}{4}} \leq \min\left\{\frac{1}{2} - \epsilon, -\frac{1}{2} + \sqrt{s^2 + \epsilon^2}\right\}.$$

Considering only the equation

$$\frac{1}{2} + s - \sqrt{3s - \frac{3}{4}} \leq \frac{1}{2} - \epsilon$$

we see that $\epsilon \leq \sqrt{3s - \frac{3}{4}} - s$. Note that $\sqrt{3s - \frac{3}{4}} - s \leq 0$ for $s \leq \frac{3-\sqrt{6}}{2}$ and increases monotonically up to $\frac{1}{2}$ over the interval $\left[\frac{3-\sqrt{6}}{2}, 1\right]$. Thus for $s \leq \frac{3-\sqrt{6}}{2}$, there is no valid ϵ. We thus restrict ourselves to the regime where $s \geq \frac{3-\sqrt{6}}{2}$ and consider the inequality

$$\frac{1}{2} + s - \sqrt{3s - \frac{3}{4}} \leq -\frac{1}{2} + \sqrt{s^2 + \epsilon^2}.$$

As this inequality is strictly weaker for larger ϵ, we may assume that ϵ takes on the largest possible value, that is $\epsilon = \sqrt{3s - \frac{3}{4}} - s$. The desired inequality is then

$$\left(1 - \left(\sqrt{3s - \frac{3}{4}} - s\right)\right)^2 \leq s^2 + \left(\sqrt{3s - \frac{3}{4}} - s\right)^2.$$

Rearranging, this is equivalent to

$$1 + 2s - s^2 \leq 2\sqrt{3s - \frac{3}{4}}.$$

As both sides are positive for $\frac{1}{4} \leq s \leq 1$, this inequality holds whenever

$$0 \leq -4 + 8s - 2s^2 + 4s^3 - s^4.$$

This occurs on the entirety of the range

$$\frac{2 + \sqrt{6} - \sqrt{2 + 4\sqrt{6}}}{2} \leq s \leq 1,$$

as desired. □

From Lemma[1] we know that if the dissimilarity of the triangle is sufficiently large with respect to the maximum distance, then the dissimilarity of the triangle discovered by the greedy algorithm is off by at most a factor of 22 from the triangle with maximum dissimilarity.
In order to address the case where the triangle discovered by the greedy algorithm is "small", we relax the problem slightly and consider the problem of the finding the triangle with maximum dissimilarity in $S(s)$, that is, we ignore the condition that all three edges of the triangle must have distance at most 1. In this relaxed problem, it is easy to see that all three points lie on the surface of $S(s)$, that is, if (x, y, z) is a point in the optimal triangle then $y^2 + z^2 = r(x)^2$. Now, by using the rotational symmetry of $S(s)$ and the symmetry about the plane $x = \frac{1}{2}$, we may further assume that one of the point of the triangle has the form $(x_1, r(x_1), 0)$ with $x_1 \leq \frac{1}{2}$ and that the other two

[1] Actually, Lemma 1 gives an approximation ration of $\frac{2}{2+\sqrt{6}-\sqrt{2+4\sqrt{6}}} \sim 1.9711$ for these large triangles.

points (x_2, y_2, z_2) and (x_3, y_3, z_3) are such that $\frac{1}{2} \leq x_2, x_3$. Further, examining the critical points of the gives that z_2 and z_3 have opposite signs. These reductions make brute force search for the optimal triangle practical. In Fig. S3 we see the resulting approximation ratio indicting that the greedy algorithm provides a rough factor 2 approximation to the maximum dissimilarity problem.

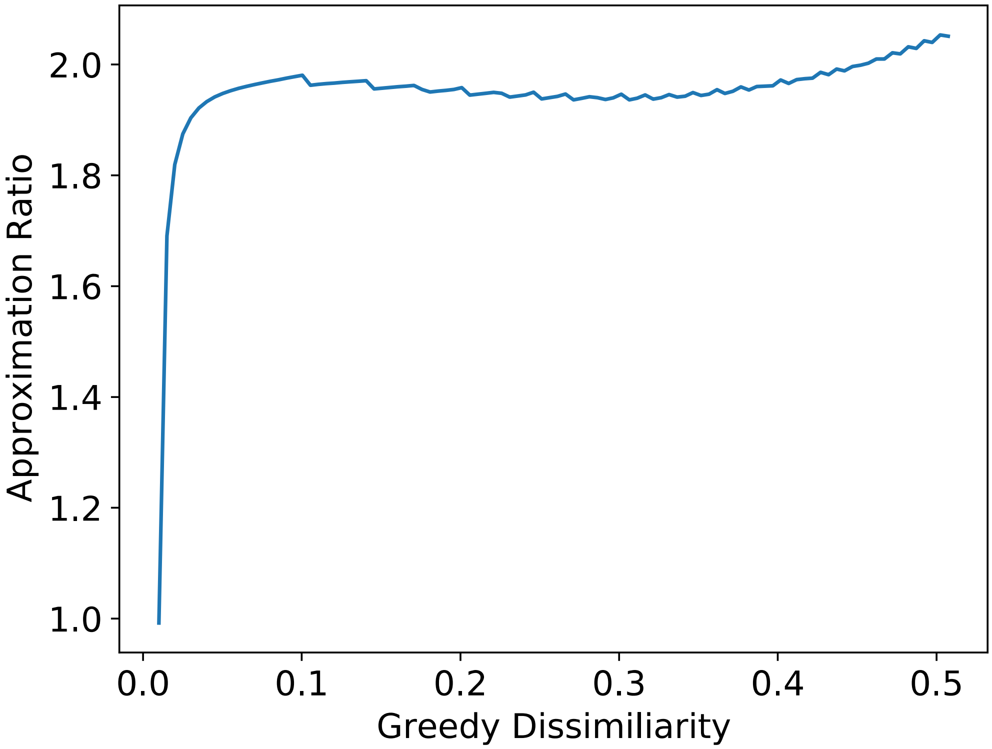

Figure. S2 Plot of the ratio between s and the triangle in $S(s)$ with the largest dissimilarity.

2. Typical Approximation Ratio

As we have seen above, the geometric constraints imposed by the solution to the greedily discovered k-clique can effectively constrain the maximum value of the dissimilarity of a point set. Unfortunately, as the geometric constraints depend on the entire history of the greedy approximation, there is little hope of easily extracting a bound on the approximation ratio based on these constraints. Thus, we instead wish to empirically estimate the maximum and average ratio between the greedy and exact solutions to the maximum dissimilarity problem for small k. To this end, we sample 200 points from 7-dimensional sphere of radius $\frac{1}{2}$ and determine the maximum dissimilarity k-clique for $k = 2, 3, 4, 5, 6$ both exactly and using the greedy heuristic. We note that even for this relatively small experiment, the naïve presents significant computational challenges with the need to consider over 8.5×10^{10} subsets of points per experiment. However, we have found that following simple observation allows for a significant trimming of the search tree.

Lemma 2. *Let X be a collection of points and let $\ell < k$. Suppose that $\{x_1, \ldots, x_k\} \subseteq X$ is a k-clique having dissimilarity at least $t^{\binom{k}{2}}$, then the ℓ-clique with maximum dissimilarity in X has dissimilarity at least $t^{\binom{\ell}{2}}$.*

Proof. Suppose not. Then, for for all $\binom{k}{\ell}$ ℓ-cliques contained in $\{x_1, \ldots, x_k\}$, the dissimilarity is strictly smaller than $t^{\binom{\ell}{2}}$. Now note that the the product of the dissimilarity of all the ℓ-cliques is at strictly $t^{\binom{\ell}{2}\binom{k}{\ell}}$. Now each distance occurs exactly $\binom{k-2}{\ell-2}$ times in this product, which gives dissimilarity of the k-clique is strictly smaller than

$$t^{\frac{\binom{\ell}{2}\binom{k}{\ell}}{\binom{k-2}{\ell-2}}} = t^{\frac{\ell!k!(\ell-2)!2!}{(\ell-2)!2!\ell!(k-\ell)!(k-2)!}} = t^{\frac{k!}{(k-2)!2!}} = t^{\binom{k}{2}},$$

a contradiction. □

As a consequence, by considering the greedy approximation (and the greedy extension of partial solutions) to finding the maximum dissimilarity k-clique, we can restrict our attention to building up from ℓ-clique which have large dissimilarity. Using this observation we are able to repeat the experiment 1000 times and estimate the maximum and typical approximation ratio for the greedy heuristic, depicted in Fig. S3. Interestingly, even though we know that the worst case approximation ratio for triangles is 2, this is unlikely to be achieved with typical approximation ratios closer to 1.1. Unsurprisingly, the quality of the greedy approximation decreases with the size of the clique, but for the typical situation still remains quite good.

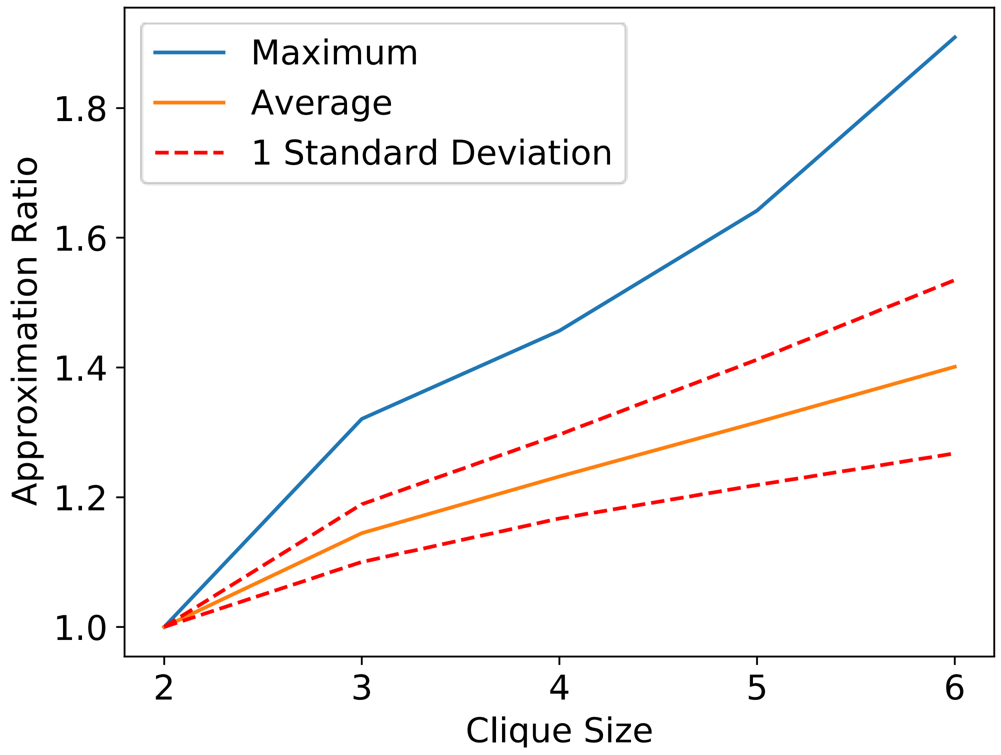

Figure S3 Approximation ratio of the greedy heuristic for 1000 trials of a set of 200 points distributed uniformly over the 7-dimensional sphere of radius $\frac{1}{2}$.

References

1. Satoshi Shimizu, Kazuaki Yamaguchi, & Sumio Masuda. A maximum edge-weight clique extraction algorithm based on branch-and-bound. *Discrete Optimization* **2020**, 37, 100583.

2. Rafael Martí, Micael Gallego, & Abraham Duarte. A branch and bound algorithm for the maximum diversity problem. *European Journal of Operational Research* **2010**, 200(1), 36-44.

3. Jay B. Ghosh. Computational aspects of the maximum diversity problem. *Operations Research Letters* **1996**, 19(4), 175-181.

4. Michael M. Sørensen. New facets and a branch-and-cut algorithm for the weighted clique problem. *European Journal of Operational Research* **2004**, 154(1), 57-70.

5. Glover, F. Improved linear integer programming formulations of nonlinear integer problems. *Management science* **1975**, 22(4), 455–460.

6. Luis Gouveia, & Pedro Martins. Solving the maximum edge-weight clique problem in sparse graphs with compact formulations. *EURO Journal on Computational Optimization* **2015**, 3(1), 1-30.

7. Hosseinian, S., Fontes, D., & Butenko, S. A nonconvex quadratic optimization approach to the maximum edge weight clique problem. *Journal of Global Optimization* **2018**, 72(2), 219–240.
